# Long-term impact of basin-wide wastewater management on faecal pollution levels along the entire Danube River

**DOI:** 10.1007/s11356-024-34190-0

**Published:** 2024-07-08

**Authors:** Alexander K. T. Kirschner, Iris Schachner-Groehs, Gerhard Kavka, Edith Hoedl, Adam Kovacs, Andreas H. Farnleitner

**Affiliations:** 1 Institute for Hygiene and Applied Immunology – Water Microbiology, Medical University Vienna, Kinderspitalgasse 15, 1090 Vienna, Austria; 2 Division Water Quality and Health, Karl Landsteiner University of Health Sciences, Dr. Karl Dorrek Straße 30, 3500 Krems, Austria; 3 Interuniversity Cooperation Centre Water & Health (http://www.waterandhealth.at), Vienna, Austria; 4 Federal Agency for Water Management, Petzenkirchen, Austria; 5 International Commission for the Protection of the Danube River (ICPDR), Vienna International Centre, Vienna, Austria; 6 Institute of Chemical, Environmental and Bioscience Engineering, Research Group Microbiology and Molecular Diagnostics, Technische Universität Wien, E166/5/3 and E057-08, Gumpendorferstraße 1a, 1060 Vienna, Austria

**Keywords:** Danube River basin, *Escherichia coli*, European Union, Faecal pollution, Joint Danube survey, Microbiological water quality, Wastewater management

## Abstract

The Danube River is, at 2857 km, the second longest river in Europe and the most international river in the world with 19 countries in its catchment. Along the entire river, faecal pollution levels are mainly influenced by point-source emissions from treated and untreated sewage of municipal origin under base-flow conditions. In the past 2 decades, large investments in wastewater collection and treatment infrastructure were made in the European Union (EU) Member States located in the Danube River Basin (DRB). Overall, the share of population equivalents with appropriately biologically treated wastewater (without disinfection) has increased from 69% to more than 85%. The proportion of tertiary treatment has risen from 46 to 73%. In contrast, no comparable improvements of wastewater infrastructure took place in non-EU Member States in the middle and lower DRB, where a substantial amount of untreated wastewater is still directly discharged into the Danube River. Faecal pollution levels along the whole Danube River and the confluence sites of the most important tributaries were monitored during four Danube River expeditions, the Joint Danube Surveys (JDS). During all four surveys, the longitudinal patterns of faecal pollution were highly consistent, with generally lower levels in the upper section and elevated levels and major hotspots in the middle and lower sections of the Danube River. From 2001 to 2019, a significant decrease in faecal pollution levels could be observed in all three sections with average reduction rates between 72 and 86%. Despite this general improvement in microbiological water quality, no such decreases were observed for the highly polluted stretch in Central Serbia. Further improvements in microbiological water quality can be expected for the next decades on the basis of further investments in wastewater infrastructure in the EU Member States, in the middle and lower DRB. In the upper DRB, and due to the high compliance level as regards collection and treatment, improvements can further be achieved by upgrading sewage treatment plants with quaternary treatment steps as well as by preventing combined sewer overflows. The accession of the Western Balkan countries to the EU would also significantly boost investments in wastewater infrastructure and water quality improvements in the middle section of the Danube. Continuing whole-river expeditions such as the Joint Danube Surveys is highly recommended to monitor the developments in water quality in the future.

**Supplementary Information:**

The online version contains supplementary material available at 10.1007/s11356-024-34190-0.

## Introduction

Large rivers are important ecosystems, connecting people over continental scales. They provide several essential ecosystem services by acting as drinking water resources, as reservoirs for recreational activities and for fishery, as resources for crop irrigation, industrial water use or as transportation routes for navigation. At the same time, rivers serve as the main recipients of anthropogenic (municipal, agricultural and industrial) wastewater with tremendous impacts on river water quality. Point sources (e.g. treatment plant effluents, raw wastewater inputs, ship wastewater discharge) or non-point sources (e.g. agricultural run-off, wild-life droppings) contribute to the chemical and microbial-faecal pollution of rivers (Damashek et al. 2022; Lee et al. 2014; Pistocchi et al. 2019).

The Danube River is, at 2857 km in length, the second longest river in Europe and the most international river in the world. Its catchment expands over an area of 803,260 km^2^ and includes 19 countries with approximately 79 million inhabitants (ICPDR 2021a). From the ten riparian countries, seven (Germany, Austria, Slovakia, Hungary, Croatia, Romania and Bulgaria) are Member States of the European Union (EU), and three (Serbia, Ukraine and Moldova) are not. Whilst Germany and Austria belong to the so called “old” EU Member States, Slovakia and Hungary joined the EU in 2004, Romania and Bulgaria in 2007 and Croatia followed in 2013. EU Member States have to comply with EU legislation concerning water quality and ecosystem health such as the EU Water Framework Directive (WFD) (European Parliament & Council 2000), the EU Urban Wastewater Treatment Directive (UWWTD) (European Council 1991) and the EU Bathing Water Directive (European Parliament & Council 2006). Due to these EU Directives, effective state-of-the-art wastewater treatment infrastructure (collection and secondary or tertiary treatment depending on the sensitivity of the receiving waters, or appropriate individual treatment facilities where centralised treatment is not feasible) must be implemented in all EU Member States to prevent further degradation of river ecosystems and to achieve improvements in river water quality. Moreover, since the Black Sea was significantly suffering from eutrophication in the late 1980s, the receiving coastal areas were designated as a sensitive area; thus, more stringent treatment technology than secondary treatment (nutrient removal) is needed at least at the medium-sized and large treatment plants in the entire DRB. When the EU WFD was adopted in the year 2000, all countries cooperating under the Danube River Protection Convention (DRPC) decided to make all efforts to implement the WFD throughout the whole Danube River Basin. Following this commitment, measures addressing adequate wastewater collection and treatment are fostered to be implemented by Non-EU Member States as well, in order to ensure a consistent development and pollution control strategy in the wastewater sector.

Measure implementation in the wastewater sector was first intensified in the upstream Danube countries (Germany, Austria) in the 1990s, when the EU UWWTD came into force. The ambitious provisions of the Directive were further reinforced and even strengthened by the EU WFD after 2000. Furthermore, the EU enlargement process around the mid-2000s and later in 2013 resulted in the accession of additional 7 Danube countries requiring substantial investments and modernization in wastewater management in order to reach higher environmental objectives. Non-EU Member States have implemented a number of investment projects in the wastewater sector in line with their national strategies.

In 2001, the International Commission for the Protection of the Danube River (ICPDR) organised the first Joint Danube Survey (JDS) with the aim of a whole-river assessment of the biological and ecological status of the Danube River according to the EU WFD, along a stretch of > 2600 km (ICPDR 2002). In the frame of this survey, also the microbiological water quality of the entire river was assessed for the first time. Patterns of microbial faecal pollution and pollution hotspots were identified from the headwaters in Germany to the Danube River Delta shortly before entering the Black Sea (Kavka and Poetsch 2002). Thereafter, the JDS was organised every 6 years in 2007 (JDS2), 2013 (JDS3) and 2019 (JDS4) (ICPDR 2008, 2015, 2021b) including the assessment of microbial faecal pollution (Kirschner et al. 2015a, 2008, 2009, 2017, 2021).

The aim of this study was to summarise the microbiological results of the four JDS in a comparative way in order to identify common patterns and trends over the nearly two decades. The data were related to progress in wastewater treatment infrastructure in all, and specifically, in the “new” EU Member States. Due to the implementation of EU legislation and advancements in wastewater treatment infrastructure, we hypothesised a significant improvement in microbiological water quality of the Danube River.

## Materials and methods

### Joint Danube surveys

During JDS1, JDS2 and JDS3, a ship with an onboard laboratory travelling down the river was provided for collecting, preparing and analysing the samples (Suppl. Inform. Fig. S1). Scientists from various biological and chemical disciplines shared a common laboratory, but a dedicated part of the work benches was reserved for microbiological analysis only, enabling setting up a sterile working environment. During JDS4, no such laboratory ship was available, and samples were collected by car and analysed in six partner laboratories in Germany (Dept. of Biochemistry III, University of Regensburg), Austria (Institute for Hygiene and Applied Immunology, Medical University of Vienna), Hungary (Dept. of Microbiology, ELTE University Budapest), Serbia (Institute of Biological Research Sinisa Stankovic, University of Belgrade) and Romania (Romanian Waters Laboratories Turnu-Severin & Calarasi).

### Sampling

Sampling for JDS1 to JDS4 took place at the Danube River and at important tributaries (rivers with catchment areas > 4000 km^2^), a few hundred metres upstream of the confluence site with the Danube. The number of samples and selected sampling sites varied over the four JDS (see Suppl. Inform. Table S1 for a detailed overview). Water samples were collected in sterile 250 to 1000 ml borosilicate glass or one-way plastic bottles from small rubber boats at a water depth of 30 cm. During JDS1 and JDS2, samples were collected only at the middle of the river, whilst in JDS3 and JDS4, samples were taken cross-sectionally, in the middle and at both river sides (approx. 15 m away from the river bank) at most of the sampling sites. The samples were stored in a cooling box in the dark and transferred to the laboratory within a maximum of 30 min (JDS1-3) or 4 h (JDS4), where samples had to be transported by car to the partner laboratories. For representative sites, replicate samples were collected for quality control purposes (see chapter “Faecal pollution analysis – Quality control”). All data used in this comparative study were taken from previous publications on faecal pollution monitoring during JDS1-4 (Kavka and Poetsch 2002; Kirschner et al. 2015a, 2015b, 2008, 2009, 2021). However, to enable a profound interpretation of the results, a brief methodical description of faecal pollution analysis is provided in the current study.

### Faecal pollution analysis

As standard indicators of faecal pollution, faecal coliforms and *Escherichia coli* were selected for the comparison of the faecal pollution levels between the four JDS. Faecal coliforms (FC) were determined in JDS1, whilst in JDS2-4 *E. coli* was determined. FC are dominated by *E. coli* populations and have been shown to occur in comparable numbers (Park et al. 2006) (Kloot et al. 2006).

#### Laboratory analysis

Prior to analysis, the working bench was disinfected with 70% ethanol, and a Bunsen burner was used to establish an aseptic working environment. All materials used were sterilised before use. Before the samples were processed, the bottles were homogenised by vigorous shaking. For the determination of FC, different volumes (1 to 100 ml) of water were filtered through 0.45 μm pore-size cellulose-nitrate membrane filters (Sartorius, Göttingen, Germany) with 50 mm diameter. The filters were placed on selective mFC-Agar (BD Difco, Fisher-Scientific, Schwerte, Germany) and incubated for 24 ± 2 h at a temperature of 44 ± 0.2 °C in sealed metal cylinders in a water bath according to ISO standard 9308–1:1990 (ISO 1990). *E. coli* concentrations were determined with Colilert 18 (IDEXX, Ludwigsburg, Germany), a most probable number (MPN) technique according to ISO 9308–2 (International Organization for Standardization 2012). Two sample volumes (100 ml and 1 ml diluted with sterile de-ionised water) were mixed with the Colilert substrate, filled into Quanti-Tray/2000 bags and sealed with a Quanti-Tray Sealer. Quanti-Trays were incubated at 36 ± 2 °C for 18—22 h and analysed in a UV-cabinet. Quantitative values were obtained by comparison with the MPN table provided by the manufacturer.

#### Quality control

During JDS1, duplicate analyses of 5% of samples and of at least one sample per test run were performed. Known positive and negative reference strains were tested for microbiological media control. No significant differences between the replicates were observed (Kavka and Poetsch 2002). During JDS2, representative parallel samples were taken and immediately brought to reference laboratories in Germany, Austria and Slovakia within a maximum of 4 h for comparative measurements via ISO standards (see Suppl. Information, Fig. S2). As ISO 9308–2 has been extensively shown to deliver comparable results with other established methods of *E. coli* quantification for different water types except drinking water (Buckalew et al. 2006; Niemela et al. 2003; Yakub et al. 2002), no such comparative measurements were performed during the other surveys.

#### Statistical analysis

IBM-SPSS, version 28, was used for all statistical analysis. Levene’s test was applied to confirm the homogeneity of variances in the data sets. For identifying differences in *E. coli* levels between the JDS, univariate ANOVA was applied with a Tukey-HSD post-hoc test. Pearson correlations were calculated to assess the similarities in the pollution patterns observed for the four JDS. A probability level of *p* ≤ 0.05 was accepted as significant.

### Assessment of the status of urban wastewater treatment in the Danube River Basin

Comprehensive inventories on urban wastewater collection and treatment systems in the DRB and their emissions discharged into the environment were developed and are regularly updated by the ICPDR. Information is collected on all agglomerations with more than 2,000 population equivalents (PE) including the size of the urban settlements, the connection rate of the population to wastewater collecting systems and wastewater treatment plants (WWTP), the treatment stage of the plants, the wastewater discharge rate and basic pollution figures. These inventories serve the pollution assessments of the Danube River Basin Management Plans elaborated by the ICPDR every six years. So far, three inventories were compiled in 2009, 2015 and 2021 for the reference years 2005/2006, 2011/2012 and 2018, respectively (source: https://www.icpdr.org/library/maps-data; accessed: June 19, 2024).

The basin-wide situation of wastewater management for the above-mentioned reference years was mapped in the framework of the three previously elaborated DRB Management Plans. These maps show the collection and treatment type of each agglomeration in the DRB, differentiating the agglomerations based on their size and the proportion of their PE connected to their collection and treatment system (> 80% or < 80%). In 2009 and 2015, the highest technological level available at a given agglomeration was chosen for the classification of the collection and treatment type, whereas in 2021, the dominant technological level was used.

In all maps, "individual and other appropriate systems (IAS)" refer to technologies such as standardised watertight storage tanks, septic tanks with infiltration fields, small domestic treatment plants, or small streatment units. In 2009, Hungary reported a special category “Collected and secondary treatment or other more stringent treatment than N and/or P removal”, indicating treatment in combination with e.g. chlorination, ozonation or sand filtration. For 2015 and 2021, this category was merged with the category “Collected and secondary treatment”. For the 2009 and 2015 maps, the class “Not collected and not treated” indicated all kinds of inappropriate individual systems for both the EU and non-EU Member States. For the 2021 map, the class “Not collected and not treated” was referring only to dry sanitation in the non-EU Member States whereas it remained the same as before for the EU Member States. In addition, the category “Local systems” (septic tanks and cesspools) was introduced for the non-EU Member States.

The development of the urban wastewater sector was evaluated by comparing the PE connected to different collection and treatment types summed up over the three main sub-regions of the DRB (Upper-DRB: Germany, Austria, Czech Republic and Slovakia; Middle-DRB: Hungary, Slovenia, Croatia, Bosnia and Herzegovina, Serbia and Montenegro; Lower-DRB: Romania, Bulgaria, Moldova and Ukraine) as well as over the entire basin.

## Results

### Consistent longitudinal patterns of faecal pollution

**Fig. 1:**
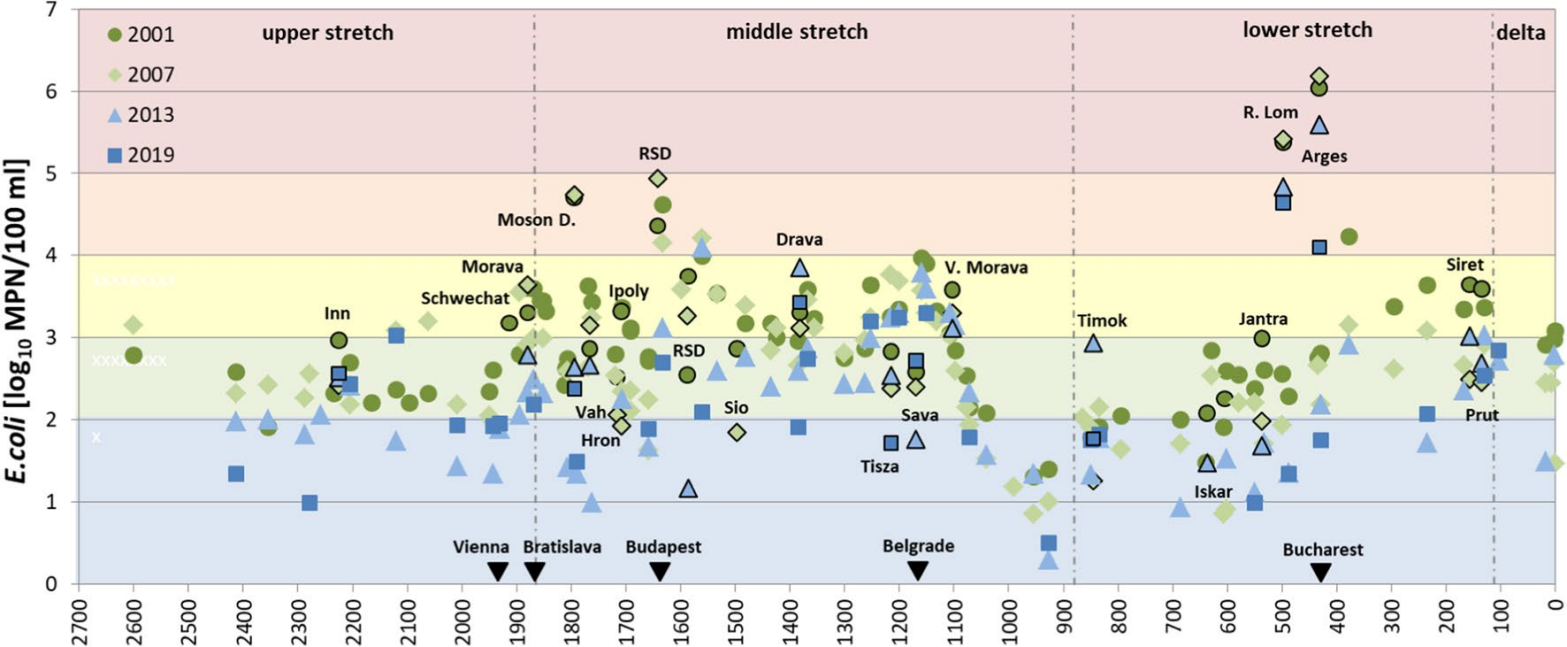
Longitudinal patterns and long-term trends in faecal pollution in the Danube River from 2001 (JDS1) till 2019 (JDS4). Black-framed symbols represent the sampled tributaries and branches of the Danube River. *Moson D*, Moson Danube; *RSD*, Rackeve-Soroksar Danube branch; *V. Morava*, Velika Morava; *R. Lom*, Rusenski Lom. The positions of the country capitals, discharging directly (Vienna, Austria; Bratislava, Slovakia; Budapest, Hungary; Belgrade, Serbia) or indirectly (Bucharest, Romania) into the Danube River are indicated with black triangles. Background colours refer to the pollution status according to Kirschner et al. (2009), red: excessive, orange: strong, yellow: critical, green: moderate, blue: little pollution

Quite consistent longitudinal patterns of faecal pollution have been observed for the four Joint Danube Surveys (Fig. 1).

**Fig. 2:**
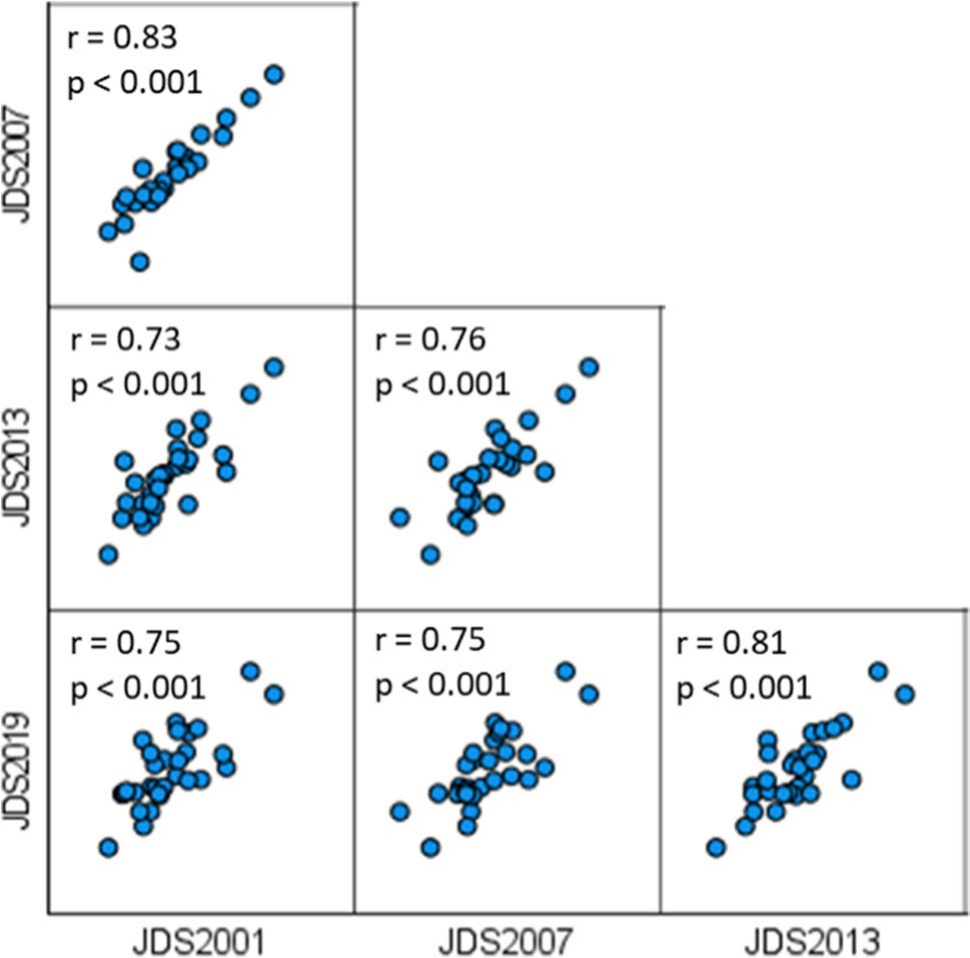
Highly significant positive correlations between the *E. coli* concentrations measured at the 28 identical sites (19 Danube River and 9 tributary sites) during the four Joint Danube Surveys, (JDS) 2001, 2007, 2013 and 2019

In the upper stretch including Germany and Austria, *E. coli* concentrations between 1 log10 and 3 log10 MPN/100 ml have been recorded at most sites. In the middle stretch, *E. coli* concentrations started to rise with maximum values > 4.5 log10 MPN/100 ml in the Moson Danube and Rackeve Soroksar Danube branches and downstream of Budapest in 2001 and 2007. Continuously high values between 3 log10 and 4 log10 occurred in the Central Serbian part of the Danube River (rkm 1250–rkm 1100), where Sava and Tisza tributaries showed lower pollution levels compared to the Danube River. Large cities such as Novi Sad and Belgrade that still do not have wastewater treatment facilities mostly contributed to river pollution in this section. Along the Iron Gate Reservoirs (rkm 1127–954), faecal pollution levels dramatically dropped due to the absence of large settlements and sedimentation processes in these large reservoirs. After the Iron Gate Reservoirs, a steady increase in faecal pollution levels in the Danube could be observed, peaking after the confluences of the highly polluted tributaries Rusenski Lom (Bulgaria, receiving wastewater from Ruse) and Arges (Romania, receiving wastewater from Bucharest). The high correspondence between the patterns of faecal pollution of the four JDS is reflected by the highly significant positive correlations between the *E. coli* concentrations of all four surveys (Fig. 2). For this comparison, only data from identical sampling sites that were sampled during all four JDS were included (*n* = 28; 19 from Danube River, 9 from tributaries).

### Long-term decreasing trend in faecal pollution levels

**Fig. 3:**
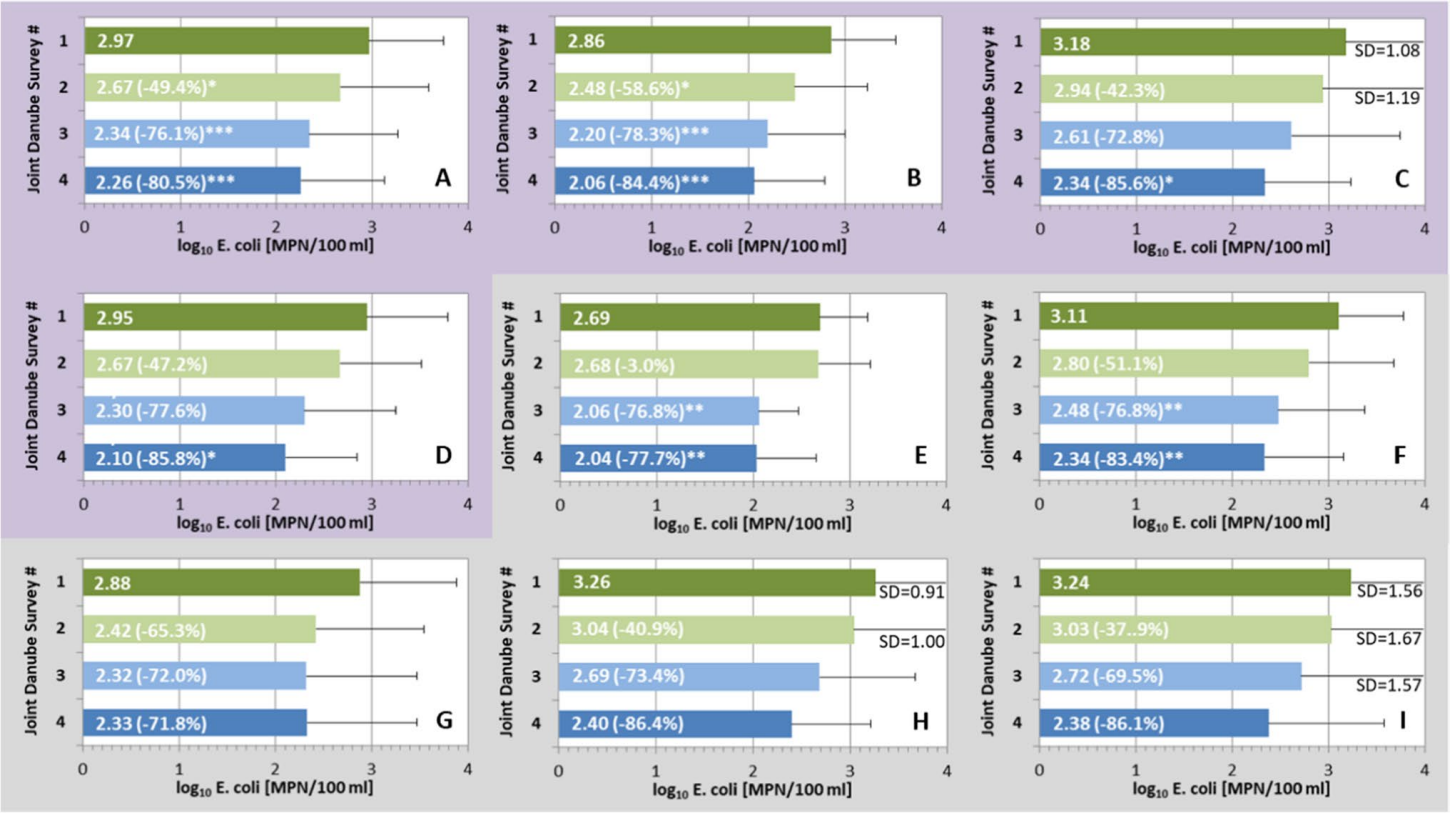
Average *E. coli* concentrations (log10 MPN/100 ml) in the respective river stretch during the four Joint Danube Surveys, JDS1 to JDS4. **A**–**D** (purple background): entire Danube River results, **A** all sampling sites at the Danube River and tributaries, **B** all Danube River sites, **C** 28 identical Danube River and tributary sites, **D** 19 identical Danube River sites; **E**–**I** (grey background): section results, **E** all Danube River and tributary sites of the upper stretch (rkm 2600–rkm 1880), **F** all Danube River and tributary sites of the middle stretch (rkm 1880–rkm 945), **G** all Danube River and tributary sites of the lower stretch including the Delta (rkm 944–rkm 0), **H** 14 identical Danube River and tributary sites of the middle stretch and **I** 9 identical Danube River and tributary sites of the lower stretch including Delta. Values in brackets indicate the % reduction in comparison to JDS1 (year 2001). *significant at *p* < 0.05; ** significant at *p* < 0.01, *** significant at *p* < 0.001; error bars represent one standard deviation (SD); when SD bars exceeded frame borders, their values were indicated

Over the whole Danube River including the most important tributaries, a clear trend towards lower faecal pollution levels from the year 2001 (JDS1, dark green symbols) to 2019 (JDS 4, dark blue symbols) was observed (Fig. 1). ANOVA revealed a statistically significant difference between the four JDS (*p* < 0.001). According to Tukey-HSD post-hoc comparisons, average *E. coli* concentrations significantly decreased from 2.97 log10 MPN/100 ml in 2001 to 2.26 log10 MPN/100 ml in 2019, corresponding to a reduction by 80.5% (Fig. 3A). A significant reduction was already observed in 2007 (− 49.4%) and 2013 (− 76.1%). Considering Danube River sites only, a similar significant reduction was recorded (Tukey-HSD post hoc comparison, *p* < 0.001), from 2.86 log10 MPN/100 ml in 2001 to 2.06 log10 MPN/100 ml in 2019, corresponding to a reduction by 84.4% (Fig. 3B). As the numbers and locations of the investigated sites were not the same for all JDS, we also ran this comparison only for those sites, which were sampled and analysed during all surveys. Also here, ANOVA revealed statistically significant differences between the four JDS (*p* < 0.05). Significant reductions from 3.15 log10 MPN/100 ml in 2001 to 2.30 log10 MPN/100 ml in 2019 were observed, when the 28 identical sites were analysed (*n* = 28, Fig. 3C), corresponding to a reduction by 85.4% (Tukey-HSD post hoc comparison; *p* < 0.05). For the Danube River sites (*n* = 19, Fig. 3D), a significant reduction by 85.9% occurred from 2.92 log10 MPN/100 ml in 2001 to 2.06 log10 MPN/100 ml in 2019 (Tukey-HSD post hoc comparison; *p* < 0.05).

We also explored whether the reductions were observed in all three different stretches of the Danube, including all Danube River and tributary sites. Interestingly, a significant decrease by 77.7% occurred in the upper stretch between 2001 and 2019 (Tukey-HSD post hoc comparison; p < 0.01; Fig. 3E). In the middle stretch, a significant reduction by 83.4% between 2001 and 2019 was observed (Tukey-HSD post hoc comparison; p < 0.01; Fig. 3F). In the lower stretch (including Danube Delta sites), a reduction by 71.8% was recorded, however, it was not statistically significant (Tukey-HSD post hoc comparison; p > 0.05; Fig. 3G). When only identical sites were included, the observed reductions were 86.4% for the middle (n = 14) and 86.1% for the lower stretch (*n* = 9) (Fig. 3H,I). Due to the low number of samples in the middle and lower stretches, the reductions were not statistically significant. For the upper section, too little data was available to allow such a comparison.

### Hotspots

Hotspots of faecal pollution were observed in the middle and lower stretches of the Danube River and its respective tributaries. Drastic reductions in faecal pollution levels were observed for some of them. At the Moson Danube (receiving the wastewater from Györ, Hungary, approx. 130.000 inhabitants), *E. coli* concentrations dropped from values around 4.5 log10 MPN/100 ml during JDS1 and JDS2 by approximately 2 log10 (100 fold!) to values around 2.5 log10 MPN/100 ml in JDS3 and JDS4. Similarly, downstream Budapest (rkm 1632) and in the RSD branch at Budapest (rkm 1588), values dropped by around 2 logs from 4.61 to 2.69 log10 MPN/100 ml and from 3.75 to 1.16 log10 MPN/100 ml, respectively (Fig. 1). In the hotspot tributaries in the lower stretch, values dropped by approximately 1 log10 from 5.4 to 4.6 log10 MPN/100 ml in Rusenski Lom (Bulgaria, rkm 498) and by approximately 2 log10 from 6.2 to 4.1 log10 MPN/100 ml in Arges (Romania, rkm 432). The decrease in the Arges River also resulted in 1 log10 decrease in faecal pollution levels at the Danube River downstream of the confluence site (rkm 329) from 2.8 to 1.8 log10 MPN/100 ml (Fig. 1). In contrast, no such consistent decreases were observed for the large tributaries in the middle section, the Drava, the Tisza and the Sava rivers. No such decreasing trends were evident for the highly polluted Danube River stretch in Central Serbia between Novi Sad and downstream Belgrade (rkm 1250–rkm 1100) with consistent *E. coli* concentrations between 3 and 4 log10 MPN/100 ml (Fig. 1).

### Development of the urban wastewater treatment status in the Danube River Basin

**Fig. 4:**
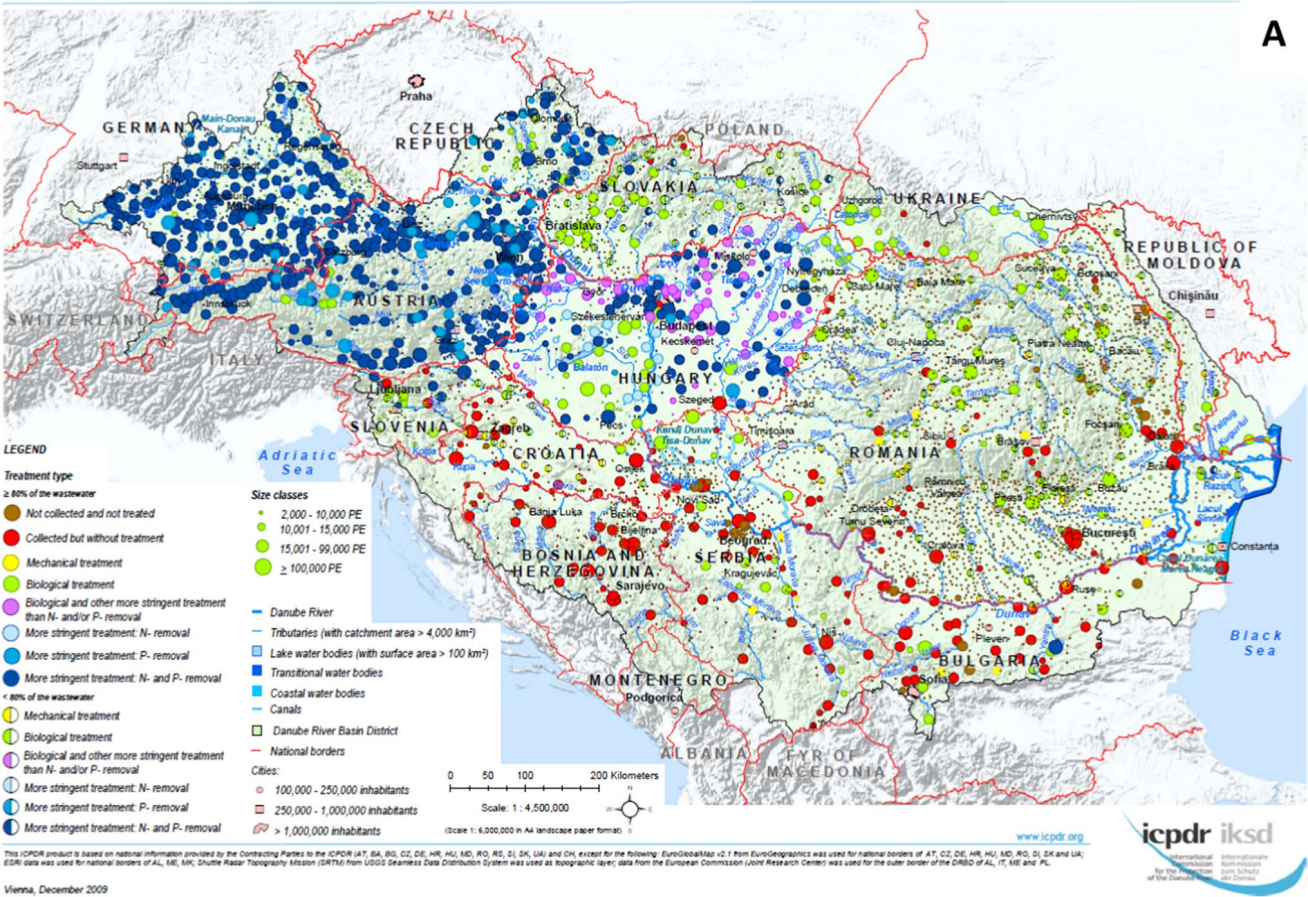

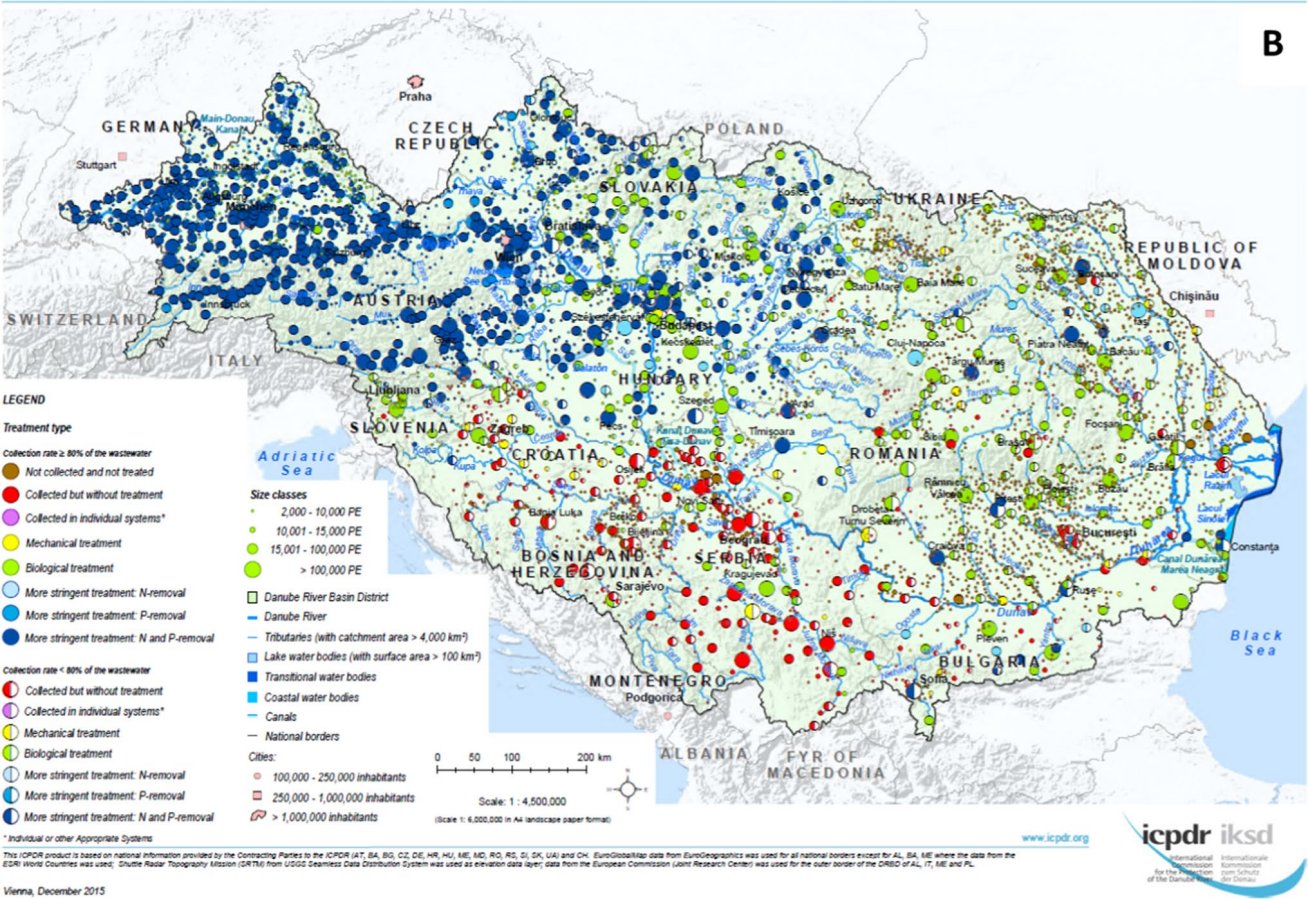

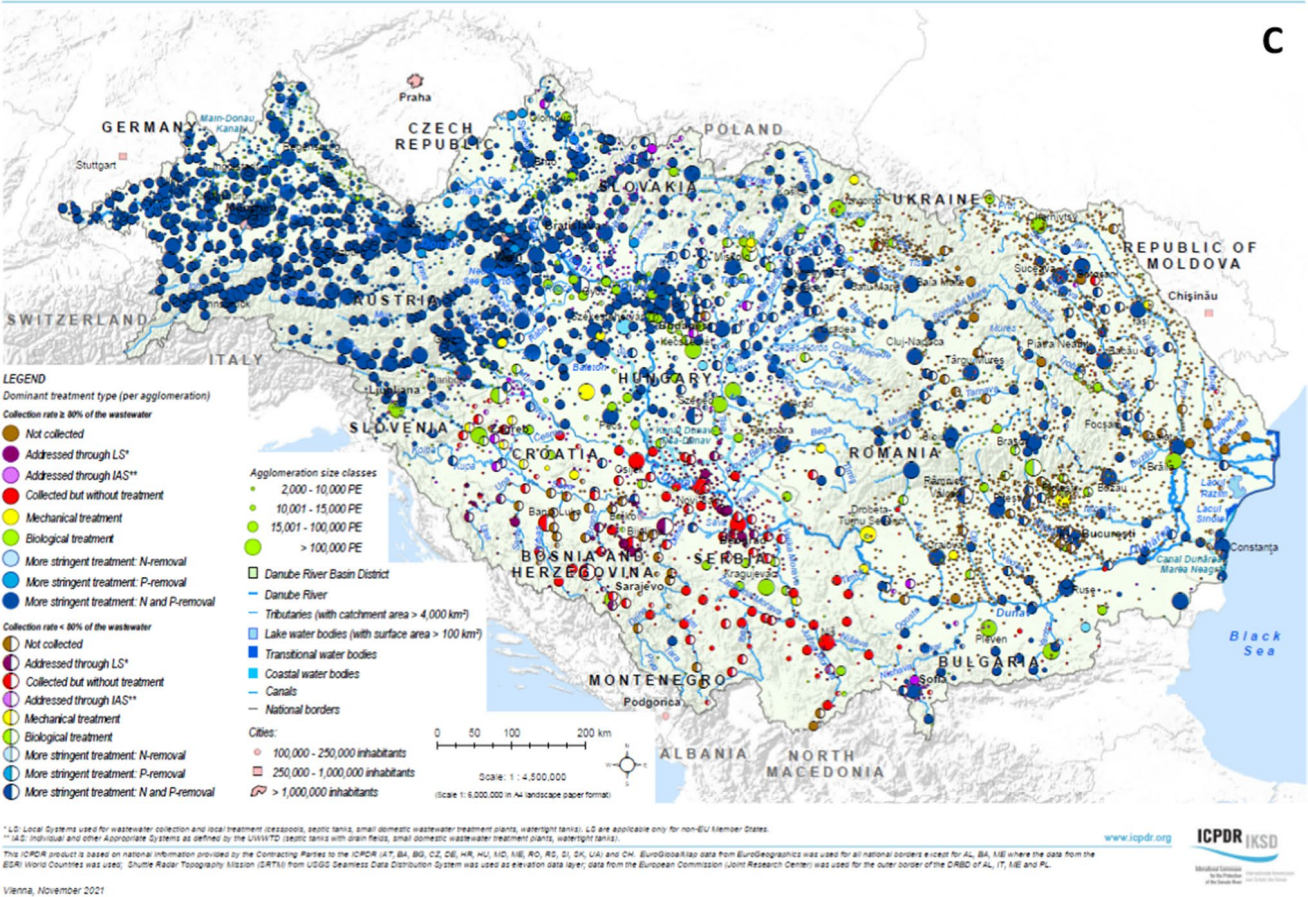
Urban Wastewater Collection and Treatment in the Danube River Basin—**A** reference situation 2005/2006, **B** reference situation 2011/2012, **C** reference situation 2018 (source: ICPDR, reprinted from [https://www.icpdr.org/] with permission from ICPDR)

Figure 4A–C shows the situation of urban wastewater collection and treatment in the DRB for three reference years (2005/2006, 2011/2012 and 2018). The 2005/2006 map shows a dual picture: whilst the Upper-DRB countries (in particular Germany and Austria) and the upstream countries in the Middle-DRB (Hungary and Slovenia) have enhanced treatment categories and inadequate technologies were in place only at small agglomerations, the remaining countries are dominated by insufficient wastewater services, largely influenced by agglomerations where wastewater is collected but not treated. Over the past decade, Danube countries have invested billions of Euros to construct collection and treatment infrastructure to improve their wastewater services, resulting in a much more balanced situation as of 2018. Only a few dots of the “Not collected” category are visible for the mid-sized and larger agglomerations whilst more scattered dots can be seen for the small municipalities. The “Collected but not treated” category is mainly confined to certain sub-regions of the basin. Green and blue colours dominate the pattern indicating the spread of high standards (tertiary and secondary treatment) except for some regions of the middle and lower stretches of the Danube River.

**Fig. 5:**
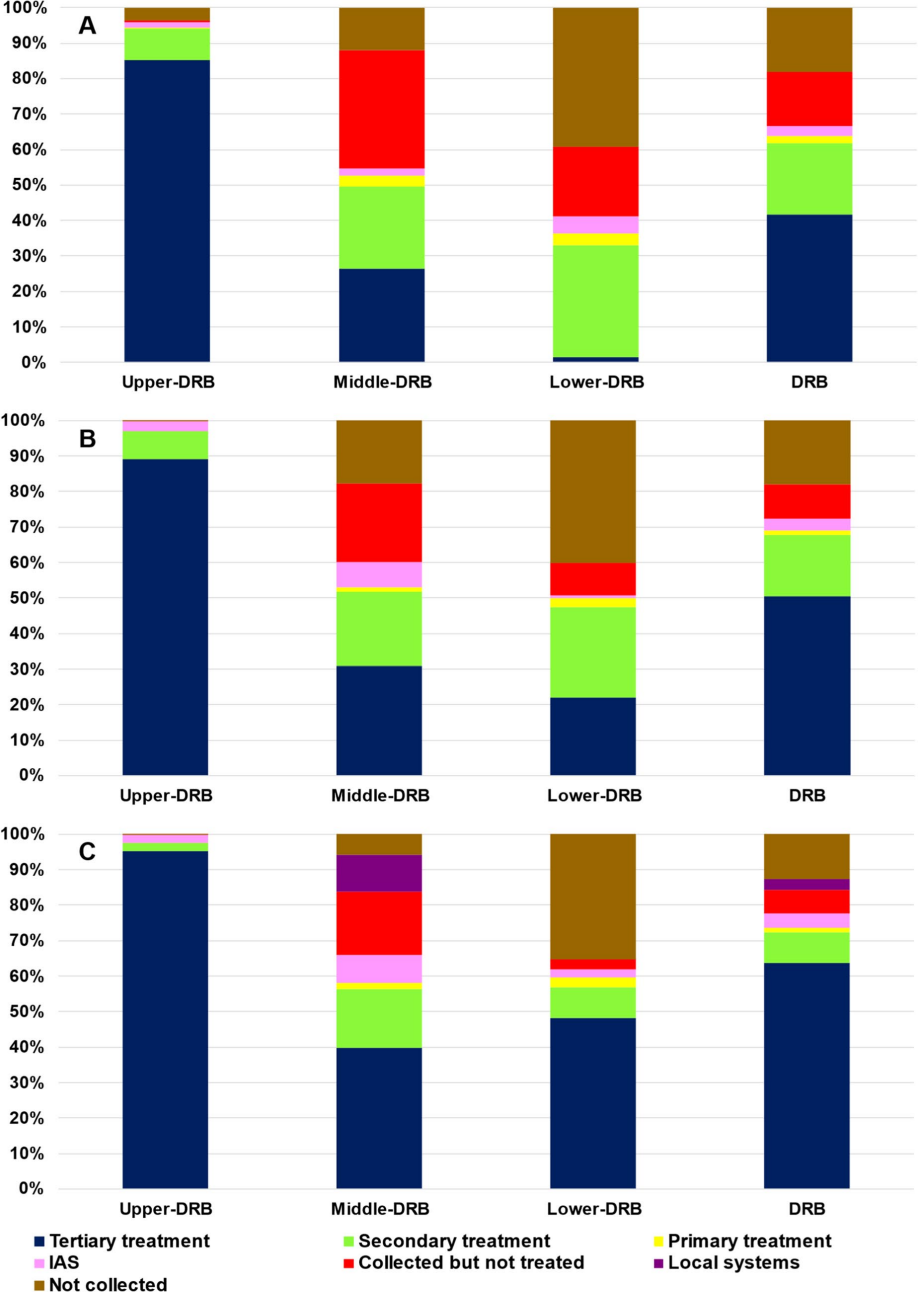
Proportion of the collection and treatment levels in the total population equivalents in the Danube countries and its sub-sections—**A** reference situation 2005/2006, **B** reference situation 2011/2012, **C** reference situation 2018 (source: ICPDR)

Figure 5 represents the proportions of the wastewater collection and treatment types for the total PE of the DRB and its upper, middle and lower stretch. In 2005/2006, only about 65% of the basin-wide PE were provided with adequate wastewater services (Fig. 5a). Wastewater was treated by (mechanical) primary treatment for 2% of the total PE, it was “Not collected” for 18%, whereas it was “Collected but not treated” for ca 15%. Regional differences were significant; whilst in the Upper-DRB high-level treatment was available for 96% of the total PE, the other regions reached only 52% (Middle-DRB) and 38% (Lower-DRB) proportions. In the Middle-DRB, the share of “Collected but not treated” wastewater was significant, whereas in the Lower-DRB wastewater was “Not collected” for a large part of the total PE. As a consequence of the substantial efforts of the Danube countries made in the wastewater management sector in the past two decades, a clear shift towards enhanced technologies can be seen from 2005/2006 towards 2018. In 2018, the majority (76%) of the overall PE of the basin was effectively treated with at least secondary treatment or addressed by adequate individual treatment facilities (IAS), whilst 24% (approx. 20 million PE) still needed basic infrastructural development in order to provide appropriate wastewater collection and treatment services (Fig. 5c). Wastewater collection and treatment systems were very much enhanced in the Upper-DRB countries with adequate technologies in place for almost 100% of the total PE, and they showed a good progress in the Middle-DRB (64%) and Lower-DRB (59%). However, significant proportions of the generated loads were still “Not collected” or “Collected but not treated” in the downstream countries of the Middle-DRB and in the Lower-DRB.

## Discussion

### Substantial improvements in wastewater treatment have led to a significant reduction in faecal pollution in the Danube River

Over the past 2 decades (2001–2019), a substantial reduction in faecal pollution levels could be recorded along the entire Danube River. On average, 80.5% lower *E. coli* concentrations were observed in 2019 in comparison to 2001, with average values dropping from 925 MPN/100 ml (= 2.97 log10) to 180 MPN/100 ml (= 2.26 log10). Interestingly, such decrease was not only observed in the middle and lower DRB, but also in the upper DRB, where tertiary and secondary wastewater treatment was already widely established in the beginning of the investigation period. Here, average values dropped from 490 MPN/100 ml (2.69 log10) to 110 MPN/100 ml (2.04 log10). The observed reduction in faecal pollution can be directly linked to the substantial progress in wastewater management, related to the implementation of the EU UWWTD (European Council 1991) in the EU Member States that requires ambitious investments in the wastewater infrastructure, even in the upper DRB. At the WWTP Vienna, Austria in the upper DRB (current load: 3.1 million PE), for example, the second biological treatment stage was put into operation in 2005. The Central WWTP in Budapest (Hungary) in the middle DRB was put into operation in 2009 (1.4 million PE), at Bucharest (Romania) in the lower DRB, the first large WWTP was opened in 2011 (1.3 million PE) with many continuous improvements. In contrast to EU Member States, development is slower in the non-EU Member States, which are lacking financial resources as well as institutional and technical capacity needed to manage the sector sustainably. One of the most striking examples is Belgrade, the capital of Serbia, a city with 1.4 million inhabitants which does not have any wastewater treatment infrastructure at all and directly discharges its untreated sewage into the Danube and Sava River. Whilst proper maintenance and optimised operation of the existing infrastructure are in focus in the Upper-DRB and in some countries in the Middle-DRB, the remaining countries still have great potential to reduce pollution of surface water bodies by constructing sewer systems, introducing at least biological treatment technology and/or applying modern local treatment facilities.

### Methodical challenges of long-term monitoring

The long-term comparison of results between the four JDS from 2001 to 2019 is associated with specific challenges. Despite the fact that the same methodology for quantifying *E. coli* was applied during JDS2 to JDS4 (Kirschner et al. 2015a, 2008, 2021), faecal coliforms were determined in JDS1 (Kavka and Poetsch 2002). As *E. coli* make up the dominant proportion of faecal coliforms (84–89%) (Kloot et al. 2006; Park et al. 2006), one could expect a slight overestimation of the JDS 1 values by 10 to 15%. However, when the same methods that we used in our study (membrane filtration to quantify faecal coliforms in JDS1 and Colilert for *E. coli* in JDS2 to JDS4) were compared to each other, *E. coli*/FC ratios > 1 were obtained, with 1.2 and 1.63 for two separate investigations (Kloot et al. 2006). This would indicate that the values from JDS1 were even underestimated and a higher reduction over the years would have occurred. As we do not have a comparative data set of the two methods for the Danube River, we kept the values obtained during JDS1 without any conversion. Moreover, an earlier study using additional data from the ICPDR TransNational Monitoring Network, confirmed the decreasing trend in faecal pollution levels in the Danube River for the period 2001 till 2007 (Kirschner et al. 2009).

Another potential source of bias may arise from the fact that the number and locations of the sampling sites among the four JDS were not identical. We, therefore, did not only compare trends and averages between the JDS by including all sampling sites, but also by using only those sites that were the same in all four JDS (“identical sites”). Since the % reduction values were of similar magnitude for both approaches (80–85%), the general interpretation of the data based on all sites remains valid.

Additional factors potentially introducing variability between the JDS are a different discharge, a different investigation period and other short-term local influences. Whilst JDS1 to JDS3 started in mid-August and lasted for 6 to 7 weeks, JDS4 began at the end of June and lasted only 3 weeks. As the overall pollution patterns were similar and highly significant correlations between the four JDS (including the last one) were observed, we do not conclude any significant influence of the different sampling dates on the results. Base-flow to mean-flow conditions prevailed during all JDS, with only short periods of time (a few days) where high water conditions could have impacted the results. Such short floods occurred only in the upper DRB and affected at most a few sampling sites. It is known that during floods faecal pollution is often elevated due to the mobilisation of faecal microorganisms from flooded agricultural areas (Aitken 2003) or combined sewer overflows (Lininger et al. 2022; Passerat et al. 2011). Due to the fact that base-flow to mean-flow conditions prevailed, specifically in the middle and lower DRB, where the major hotspots of faecal pollution were observed, we think that the potential influence of discharge during the JDS was negligible. Differences between the JDS due to local short-term influences cannot be ruled out, but this concerns only a few sampling sites and does not affect the overall trends. For example, such local influences were detected during JDS3 at two sites in the upper DRB, both downstream of two shipping piers in Germany and Austria, where we hypothesised that tourist ships may be discharging improperly treated effluent (Kirschner et al. 2017). No similar observations were made during the other JDS expeditions.

### Current state-of the art wastewater treatment does not result in the absence of faecal emissions

Whilst investments in wastewater treatment infrastructure in the middle and lower DRB have a high potential for further reducing faecal pollution levels, further progress in the Upper-DRB can – due to the very high compliance with relevant wastewater collection and treatment levels – only be achieved when advanced treatment strategies and/or the construction of separate sewer systems and retention basins are implemented. Current state-of-the-art wastewater treatment (i.e. tertiary treatment) does not reduce faecal pollution levels to zero. Depending on the applied technology, reduction efficiencies of faecal indicators range from 2 log10 (99%) (Mayer et al. 2016) to 6 log10 (99.9999%) (Van den Akker et al. 2014) and treated wastewater has to be regarded as highly infectious agent containing bacterial, viral and protozoan pathogens (Dias et al. 2019). Moreover, high loads of faecal pollution enter rivers during periods of heavy rainfall events, which are likely to increase in the next decades due to changes in precipitation patterns caused by the global climate crisis (Trenberth 2011). Combined sewer overflows (CSO) can account for a substantial proportion of total faecal pollution in rivers (Mascher et al. 2017; Passerat et al. 2011), impairing advancements in wastewater treatment infrastructure at least during certain periods. Heavy rainfall also leads to increased surface run-off from (manured) agricultural fields and pastures, leading in combination with CSOs, to significantly elevated faecal pollution levels during rainfall events (Aitken 2003; Lee et al. 2014). Additional sources of faecal pollution in large rivers such as the Danube are wildlife droppings (e.g. from waterfowl) or the increasing number of ships (freight ships, tourist ships). Most recently, we could show that the faecal pollution potential of the shipping industry on the Danube River in Austria can be as high as the load of the municipal wastewater treatment plant effluents, if the ships´ sewage is not treated properly (Steinbacher et al. 2024). Although we did not find evidence that the ships contributed to the faecal pollution levels in the investigated 230 km stretch in Austria, local influences below shipping stations were found (Steinbacher et al. 2024).

### Future developments and visions

With the ongoing implementation of wastewater collection and treatment infrastructure, we expect a further general decrease in faecal pollution levels in the Danube River, specifically in the EU Member States in the middle and lower DRB sections. Still existing hotspots such as the confluence sites with the Arges River (receiving the discharge of Bucharest, Romania) or Rusenski Lom (receiving the discharge of Ruse, Bulgaria) will hopefully disappear in the next one or two decades. A particular focus and priority in improving water quality should be directed towards the intensively polluted Central Serbian stretch from upstream of Novi Sad to downstream of Belgrade and the large tributaries Drava, Tisza and Sava. In this regard, the accession of the Western Balkan countries to the EU would also significantly boost investments in wastewater infrastructure to improve the water quality and reach environmental objectives in the middle section of the Danube.

In the upper DRB, where a high compliance level of wastewater collection and tertiary treatment is state-of-the-art, further improvements can only be achieved by the implementation of advanced treatment such as ozonation, advanced oxidation, UV disinfection and chlorination or by the prevention of CSOs. Recently, Demeter and colleagues (Demeter et al. 2021) have modelled future scenarios for drinking water production along the Danube River under the light of climate change and population growth. The outcome of this study was that the combination of both advanced treatment (e.g.: UV treatment) and CSO prevention (via retention reservoirs or any form of green infrastructure) was most effective, being able to reduce medium and maximum pathogen loads (norovirus, enterovirus) in river water by 4 log10, in comparison to the reference situation. Such scenarios are not unrealistic, since the new proposal for revision of the EU UWWTD (European Commission 2022) would require EU Member States to better control storm-water overflows and urban run-off as well as to implement advanced treatment at facilities > 100,000 PE or even at smaller agglomerations where micro-pollutant pollution poses a risk to human health or the environment.

The proposed revision of the EU UWWTD would also request from EU Member States to set up a wastewater surveillance monitoring program for relevant public health parameters such as SARS-CoV-2, antimicrobial resistance and other pathogens (European Commission 2022). In addition to the knowledge gained on the epidemiological situation concerning specific pathogens, such surveillance monitoring programs will lead to a higher awareness of problems associated with faecal pollution in the emitted wastewater and also in the river water. A conceptual framework for faecal microbiological pollution analysis and management has been suggested for the Danube River, which includes—next to monitoring of faecal pollution—genetic microbial source tracking (MST) of microbial faecal pollution and health risk assessment (Kirschner et al. 2015b). This framework is able to link public health with wastewater surveillance and river water monitoring (Demeter et al. 2023). For example, the application of genetic MST during JDS 2013 and JDS 2019 revealed municipal (human) faecal pollution as the main contributor to microbial faecal pollution (Kirschner et al. 2017). Large river monitoring surveys such as the JDS combined with state-of-the-art microbiological pollution diagnostics are thus ideal operations to assess the effects of the improvements in wastewater technology and infrastructure on river water quality and public health.

## Conclusions

The observed improvement of microbiological water quality along the whole Danube River impressively reflects the large investment in wastewater treatment infrastructure in the EU Member States within the last 2 decades. To demonstrate this development, long-term investigations of pollution patterns and trends are necessary. The Joint Danube Surveys, which have been performed every 6 years since 2001 are worldwide unique whole-river expeditions providing a comprehensive database for this purpose and shall be continued in 2025. To the best of our knowledge, no comparable long-term monitoring studies of other large rivers in the world exist in the scientific literature. This study can thus act as a model for other river basins stimulating research on this highly relevant topic.

### Supplementary Information

Below is the link to the electronic supplementary material.

Supplementary file1 (DOCX 997 KB)

## Data Availability

All data supporting the findings of this study are available within the paper and its Supplementary Information with exception of the data shown in the urban wastewater collection and treatment maps. All faecal pollution data are available in visualised form in the manuscript or in the Supplemental Information and can be found at the Danubis database (https://danubis.icpdr.org/). All data in the urban wastewater collection and treatment maps are available in visualised form in the manuscript or can be obtained upon request by the ICPDR (www.icpdr.org).
