# Supplementary material for: Long-term impact of basin-wide wastewater management on faecal pollution levels along the entire Danube River
Source: Environ Sci Pollut Res Int. 2024 Jul 8;31(33):45697–710. doi: 10.1007/s11356-024-34190-0 (PMC11269416; doi:10.1007/s11356-024-34190-0)
Supplement: Supplementary file 1 — Supplementary file1 (DOCX 997 KB) [file 11356_2024_34190_MOESM1_ESM.docx]

## SUPPLEMENTAL INFORMATION

# Long-term impact of basin-wide wastewater management on faecal pollution levels along the entire Danube River

Alexander K.T. Kirschner, Iris Schachner-Groehs, Gerhard Kavka, Edith Hoedl, Adam Kovacs & Andreas H. Farnleitner

**
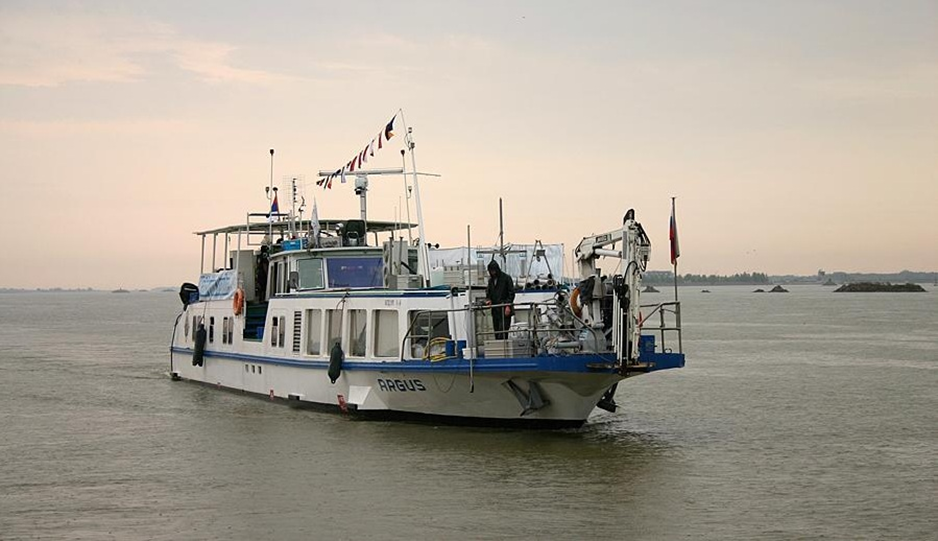
**

© ICPDR 2013

**
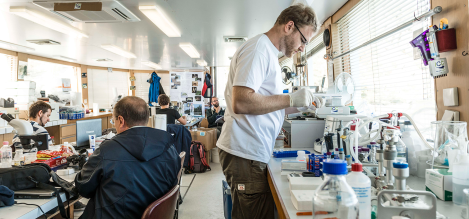
**

© ICPDR 2013

**Figure S1**: Laboratory ship „Argus” and microbiological work bench used from JDS1 to JDS3

**Table S1:** Basic Information on the Joint Danube Surveys (JDS)

| JDS | start | end | # sampling sites | # Danube sites | # tributaries  / branches |
| --- | --- | --- | --- | --- | --- |
| JDS1 | Aug 13, 2001, rkm 2581 | Sep 19, 2001, rkm 3 | 98 | 74 | 24 |
| JDS2 | Aug 13, 2007, rkm 2600 | Sep 26, 2007, rkm 0 | 96 | 72 | 24 |
| JDS3 | Aug 13, 2013, rkm 2581 | Sep 25, 2013, rkm 18 | 68 | 53 | 15 |
| JDS4 | Jun 30, 2019, rkm 2415 | Jul 19, 2019, rkm 104 | 36 | 28 | 8 |


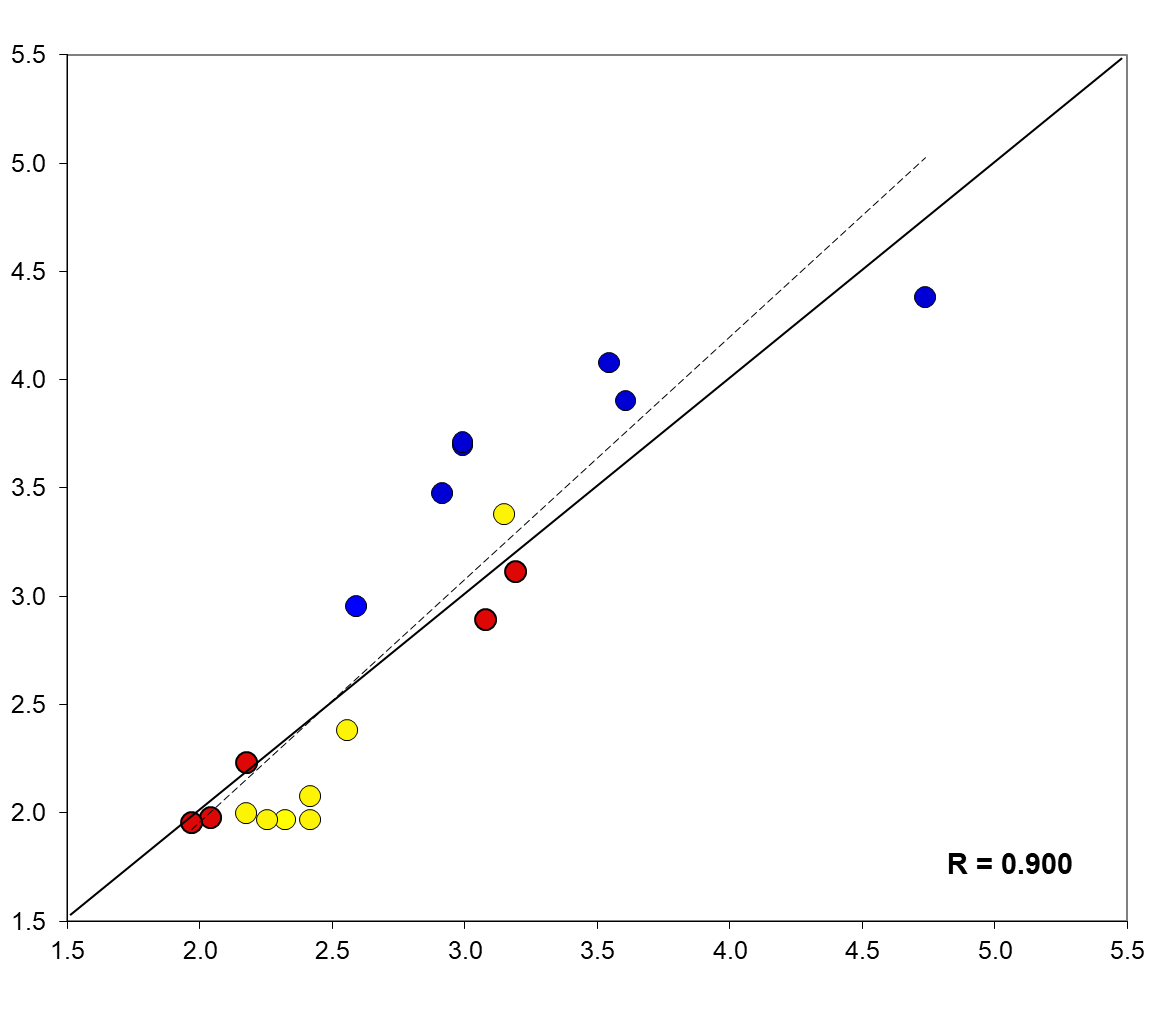


***E. coli/*FC REFLAB**

**[log MPN/CFU 100ml^-1^]**

***E. coli* ONBOARD (log MPN 100 ml^-1^)**

**Figure S2:** Comparison of the *E. coli* determination via Colilert on board of the laboratory ship with the results from the reference laboratories. **Yellow:** German reference laboratory (Bayerisches Landesamt für Umwelt, Augsburg), Faecal Coliform (FC) detection via MPN (Bundesgesundheitsblatt 10/1995, EN ISO 9308-2: 1990); **Red:** Austrian accredited reference laboratory (Institute for Hygiene and Applied Immunology – Water Hygiene, Medical University of Vienna), membrane filtration on TBX Agar (adapted from EN ISO 16649-1). **Blue:** Slovakian reference laboratory (National Water Reference Laboratory, Water Research Institute, Bratislava), membrane filtration (EN ISO 9308-1). The dashed line indicates the regression line and the solid line the 1: 1 relationship. A highly significant correlation between the data was achieved (R = 0.90, p < 0.001, n = 19) and the regression line is close to the 1: 1 relationship, indicating that the data obtained on board with the chosen methodology yielded highly comparable data of *E. coli* detection.
